# Structural changes induced by acidic pH in human apolipoprotein B-100

**DOI:** 10.1038/srep36324

**Published:** 2016-11-08

**Authors:** José A. Fernández-Higuero, Asier Benito-Vicente, Aitor Etxebarria, José Carlos G. Milicua, Helena Ostolaza, José L. R. Arrondo, Cesar Martín

**Affiliations:** 1 Biofisika Institute (UPV/EHU, CSIC), University of the Basque Country, UPV/EHU, Spain, Apdo. 644, 48080 Bilbao, Spain; 2 Dpt. Biochemistry and Molecular Biology, University of the Basque Country, UPV/EHU, Spain, Apdo. 644, 48080 Bilbao, Spain

## Abstract

Acidification in the endosome causes lipoprotein release by promoting a conformational change in the LDLR allowing its recycling and degradation of LDL. Notwithstanding conformational changes occurring in the LDLR have expanded considerably, structural changes occurring in LDL particles have not been fully explored yet. The objectives of the present work were to study structural changes occurring in apoB100 by infrared spectroscopy (IR) and also LDL size and morphology by dynamic light scattering (DLS) and electron microscopy (EM) at both pH 7.4 and 5.0. We determined by IR that pH acidification from 7.4 to 5.0, resembling that occurring within endosomal environment, induces a huge reversible structural rearrangement of apoB100 that is characterized by a reduction of beta-sheet content in favor of alpha-helix structures. Data obtained from DLS and EM showed no appreciable differences in size and morphology of LDL. These structural changes observed in apoB100, which are likely implied in particle release from lipoprotein receptor, also compromise the apoprotein stability what would facilitate LDL degradation. In conclusion, the obtained results reveal a more dynamic picture of the LDL/LDLR dissociation process than previously perceived and provide new structural insights into LDL/LDLR interactions than can occur at endosomal low-pH milieu.

LDL receptor (LDLR) is responsible for the uptake of LDL particles into cells^1^ upon binding to apoB100, the integral structural apolipoprotein for LDL particles^2^. Once internalized, the LDLR-lipoprotein complex traffics to endosomes, where the lipoprotein cargo is released and the receptor is recycled back to the cell surface^3^,^4^. ApoB100-LDLR interaction disappears in the late endosome as a consequence of acidification of this compartment^5^. Dissociation of LDL from LDLR in the endosome is a key process that enables receptor recycling and LDL hydrolysis in the lysosome^3^,^4^.

Many studies have been conducted on the LDLR to understand the mechanism of pH-dependent LDL release. It has been shown that acidification in the endosome causes lipoprotein release by promoting a conformational change in the LDLR from an extended conformation at neutral pH to a compact conformation at acidic pH^4^,^6^. Elongated stick-like LDLR structures have been observed in reconstituted vesicles at neutral pH^6^, whereas crystallographic studies at acidic pH shows a closed conformation structure of the LDLR with intramolecular contacts between cysteine-rich repeats LR4–LR5 repeats of the ligand binding domain and the β-propeller^4^. The closed conformation adopted by the LDLR at acidic pH leads to a weakened interaction with lipoproteins^7^. Although knowledge on LDLR conformational changes occurring in the cargo releasing process has expanded considerably, structural changes occurring in LDL particles have not been explored yet. In fact, acidic pH may alter the conformation of apoB100, the characteristics of the LDL particle or both, contributing thus to the cargo releasing process. It has been suggested that apoB100 is composed of globular domains connected by flexible chains that stabilize the structure of the protein-lipid complex and analysis of the sequence suggests that apoB100 contains five distinct alternating α-helical and β-sheet domains: NH- βα1-β1-α2-β2-α3-COOH with different lipid binding affinities^8^,^9^,^10^,^11^. Some regions of the apoB100 rich in β-type structures are embebded in the phospholipid monolayer of the particle^2^,^12^,^13^, while the residues involved in LDLR binding are exposed to the medium^12^. A low resolution model of LDL at extracellular pH showing the distribution of apoB100 α-helix and β-sheet rich domains across LDL surface has been determined by electron cryomicroscopy (cryoEM), a technique that preserves the native structure of the particles^14^. The modular nature of the protein, with ordered domains connected by flexible linkers has been determined by small angle neutron scattering of lipid-free apoB100^15^; modeling of the LDL core has been resolved by small-angle X-ray scattering^16^ and single particle reconstruction has been done by cryomicroscopy^17^.

Based on the above, the main objective of the present study was to evaluate the structural changes in apoB100 and lipoprotein particle occurring upon pH acidification, mimicking the acidic environment of the endosome. Therefore, we analyzed LDL size and morphology by dynamic light scattering (DLS) and electron microscopy, respectively. Infrared spectroscopy (IR) has been used to determine both secondary structure content apoB100 and LDL stability, both at pH 7.4 and 5.0. The results show differences in the apoB100 secondary structure content among lipoproteins incubated at pH 7.4 or at pH 5.0 that could resemble those occurring in the early or late endosomes, respectively. In addition, thermal stability of LDL at pH 5.0 is lower than at pH 7.4. To our knowledge this work presents a new approach for understanding the LDLR/LDL releasing process occurring upon endosomal acidification.

## Materials and Methods

### Isolation of LDL

This study was approved by the Research Ethic committee from the University of the Basque Country (Comité de Ética en la investigación y la práctica docente de la Universidad del País Vasco/Euskal Herriko Unibertsitatea; CEID/IIEB). CEID/IIEB ethics committee waived the need for written informed consent because the samples were fully anonymized. All experiments were carried out in accordance with relevant guidelines and regulations. Low-density lipoproteins were isolated from blood samples collected from sample donors using a two-step centrifugation according to published procedure^18^. Briefly, human plasma was obtained by centrifugation for 30 min 12,000 × g at 4 °C of blood samples collected in EDTA tubes. The density of serum was adjusted to 1.21 g/mL by the addition of potassium bromide, then PBS was carefully added, resulting in two phases. The sample was centrifuged by isopycnic ultracentrifugation at 244,500 × g for 19 h 30 min at 4 °C with the aim to form a density gradient along the tube, allowing the isolation of different lipoprotein particles based in their different density properties. The characteristically orange band corresponding to LDL was recovered and stored at 4 °C.

### LDL size determination

LDL size was determined from 1 mg/mL protein samples by dynamic light scattering in a Nano-S Zetasizer (Malvern Instruments, UK) as previously described^19^. For the study, all DLS measurements were performed at 37 °C in triplicate, with 25 runs of 10 seconds each, using a 173° backscatter detection. The detection limit of the assay for zetasizer instrument used in the present study was 0.3 nm to 10 μm. Viscosity and refractive indexes of PBS as the dispersant were applied to standard operating protocol prior to size determination. The data were analyzed by zetasizer family software.

### LDL size determination by negative stain electron microscopy (NS-EM)

For negative stain electron microscopy (NS-EM) a 10 μL drop of lipoprotein solution (100 μg/mL) was placed on a glow-discharged thin carbon-coated 300-mesh copper grid (Cu-300CN; Pacific Grid-Tech, San Francisco, CA). After ~ 1 min, the excess solution was removed by blotting with filter paper. The grid was washed three times by briefly touching the surface of the grid with a drop (~30 μL) of deionized water on Parafilm and then blotted dry with filter paper. The touching and blotting steps were performed, each with a clean drop of deionised water. One drop (~30 μL/drop) of 1% (w/v) uranyl acetate (UA) (pH 4.6) solution were applied on Parafilm, and the excess solution was removed by blotting similarly. The grid remained in contact with the last UA drop with the sample side down for 1–3 min in the dark before excess stain was removed and the sample was air dried at room temperature.

Particle size was determined by measuring Feret diameter, individual particle images were selected, picked automatically and manually checked to remove overlapping or damaged particles. More than 1600 particle images from micrographs of each condition were used for statistical analysis of particle size distribution.

### pH adjustment and sample preparation

Repeated concentration and dilution steps with PBS buffer made in D_2_O and adjustment at diferent pD (from 7.4 to 5.0 at the intervals indicated in the figures) were done using Microcon 100 (Millipore) filters. The H_2_O-D_2_O exchange was followed by the analysis of infrared spectrum of withdrawn buffer. This same buffer was used to determine sample pH of samples during solvent substitution process. Finally, LDL samples were concentrated to approximately 10 mg/mL. The protein content of LDL was determined according to Lowry protein assay^20^ with bovine serum albumin (1 mg/mL) as standard.

### Infrared Measurements

Measurements were performed in a Nicolet Nexus 5700 spectrometer equipped with a MCT detector. Samples were applied on a 25 μm carved calcium fluoride windows and placed in Peltier cell (TempCon, Bio Tools). Each spectrum with a nominal resolution of 2 cm^−1^ was obtained by the collection of 370 interferograms at 20 °C and then referred to a background. Then, samples were heated continuously to final temperature of 80 °C at one degree per minute speed. Simultaneously to the temperature increase Rapid Scan software from OMNIC (Nicolet Corp., Madison, USA) was used to collect double-side interferograms, which were averaged automatically after each minute. Thereby, consecutive spectra in the 20–80 °C interval correspond roughly to a temperature difference of 1 degree.

### Spectral analysis

Quantitative information on the amide I, located between 1700 and 1600 cm^−1^ and mainly composed (~80%) by the C=O stretching vibration of the peptide bond, was obtained as previously described^21^,^22^. The spectra were digitally subtracted using a spectrum of the last dialysis buffer as a reference for each sample. With the aim of minimize the differences in protein concentration among recorded samples, all spectra were normalized to amide I band area. A narrowing process that preserves the assignment of components has been used as previosly described 31. These difference spectra were analyzed by Fourier deconvolution (bandwidth = 18 and k = 2) and Fourier derivation (power = 3 and breakpoint = 0.3) in order to define the number and position of constituent bands of the amide I band. The baselines of normalized spectra were removed before starting the fitting procedure. A two steps fitting method was carried out, in which four parameters are considered for each component: band position, band height, bandwidth, and band shape. Initial bandwidth were previously estimated from the Fourier derivative and Gaussian band shape was considered for all component bands^13^. This procedure consisted in a first iterative stage in which the positions of the component bands obtained by band narrowing techniques were fixed, allowing the estimation of their final widths and heights, and a second stage where band positions were left to change. To enhance the accuracy of the comparison of amide I band decompositions, the fittings were performed using, as initial positions, the average wavenumber of each component band obtained from all LDL samples. Assignments for apoB100 amide I band components have been made as previously described^13^: α-helix (1656 cm^−1^), β-sheet (1631 cm^−1^), β-turns (1668 and 1680 cm^−1^) and unordered structures (1645 cm^−1^). The 1617 cm^−1^ band is assigned to β-strands embedded in the lipid monolayer with a high-frequency component at 1693 cm^−1^ 23.

### Statistical analysis

All measurements were performed at least 3 times and levels of significance were determined by a two-tailed Student’s t-test. A value of P < 0.05 was considered statistically significant (*P < 0.05; **P < 0.01; ***P < 0.001). The coefficient of variation for DLS was 2.3%; for EM-NS was 2.1 and for IR was 5.9%.

## Results

### LDL morphology and size are not affected by acidic pH

**Figure 1:**
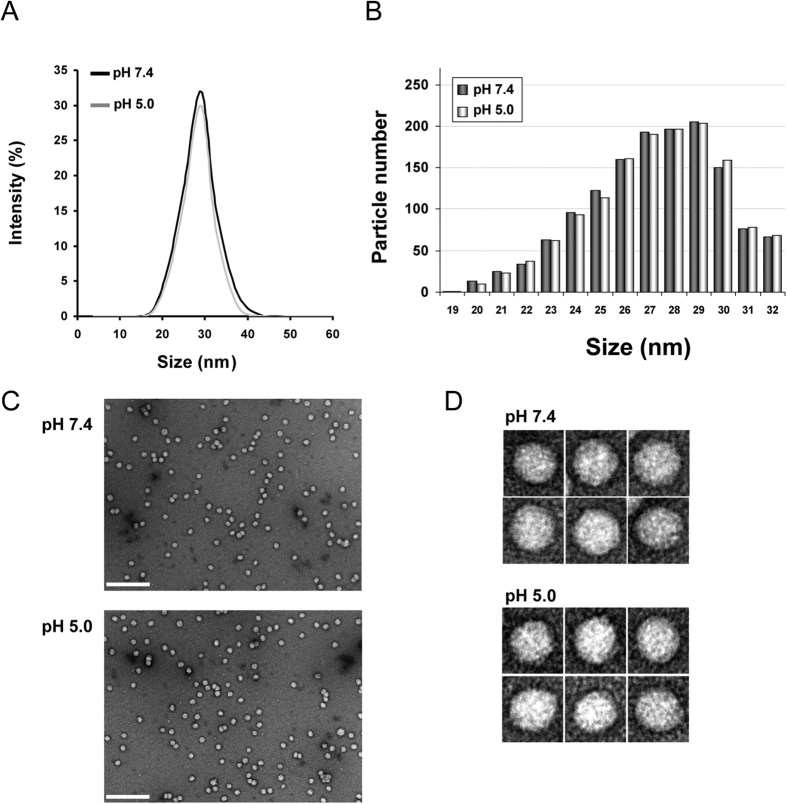
LDL size distribution and morphology at pH 7.4 and 5.0. (**A**) Size distribution of LDL at pH 7.4 and 5.0 determined by DLS; (**B**) Frequency histograms showing particle size distribution of LDL at pH 7.4 and pH 5.0. (**C**) electron-micrographs of LDL at pH 7.4 and 5.0 at low resolution showing a homogeneous particle population; (**C**) selected individual LDL particles at higher magnification. Particle size was determined as described in Materials and Methods. LDL size distribution in (**D**) was measured as Feret diameter calculated from 1600 particles.

In order to analyze changes of LDL that would facilitate particle release from its receptor LDL size was analyzed by DLS at pH 7.4 and pH 5.0 as representative of physiological and endosomal enviroments, respectively. The mean particle diameter obtained at neutral pH was 29.4 ± 0.2 nm in concordance as previously described^24^. Figure 1A shows there was no alteration of the mean particle diameter at pH 5.0, as 29.8 ± 0.4 nm value was obtained. Moreover, size distribution was similar for both experimental conditions, which discards an aggregation or degradation of LDL particle induced by acidic pH.

LDL morphology was examined by NS-EM at pH 7.4 and pH 5.0, all the particles examined in the images were approximately circular, consistent with a spherical shape (Fig. 1B,C). For LDL at both pH 7.4 and pH 5.0 the peak population of the selected 1400 particles was in the diameter range of 28–30 nm confirming values obtained by DLS (Fig. 1D).

### LDL particle undergoes reversible changes in its apoB100 structure upon pH acidification

**Figure 2:**
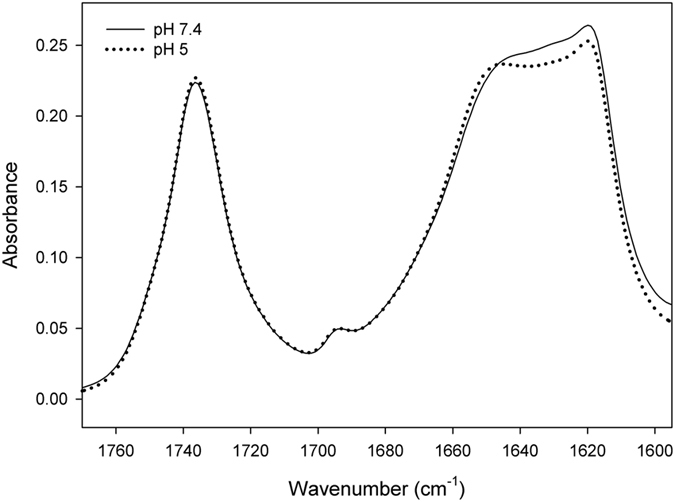
Infrared spectra of the LDL at different pHs. Infrared spectra from 1780 to 1600 cm^−1^ of LDL samples, corresponding to the absortion of lipid esters and peptide bond, were recorded in D_2_O buffer at 37 °C as described in Materials and Methods.

**Figure 3:**
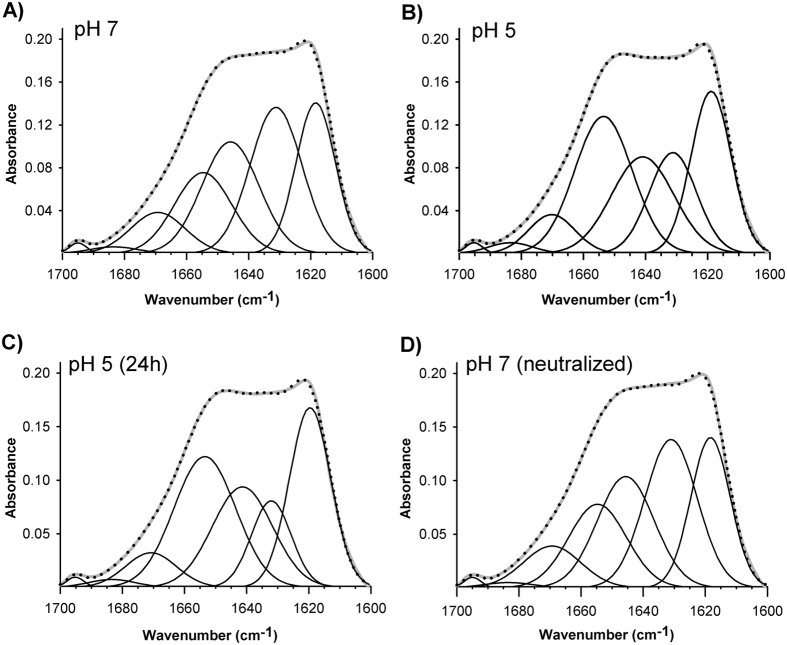
Amide I band decomposition of LDL. (**A**) pH 7.4, (**B**) pH 5.0, (**C**) LDL incubated for 24 h at pH 5.0 and (**D**) LDL after pH neutral restitution. The spectra were obtained in D_2_O buffer at 37 °C and data processed as described in Materials and Methods.

Next we explored possible changes in lipid moiety and apoB100 secondary structure by comparison of their infrared spectra taken at pH 7.4 and pH 5.0. The carbonyl vibration arising from the lipid esters, i.e. triacylglycerol, cholesterol esters that constituted lipid moiety of the particle give an absorbance band between 1780–1700 cm^−1^ 25. As shown in Fig. 2, this band was not altered after buffer acidification indicating that lipids were not affected. However, the shape of apoB100 amide I band, located from 1700 to 1600 cm^−1^ changed with a clear absorbance decrement between 1645 and 1620 cm^−1^ (Fig. 2) indicating significant changes in the secondary structure of apoB100. Figure 3 shows decompositions of apoB100 amide I band at pH 7.5, pH 5.0 and after neutral pH restitution (5.0 → 7.4). In agreement with secondary structure content determined by IR described for apoB100, at pH 7.4, β-structure is the principal conformation present in apoB100 (β-sheets ~28%, β-strands ~22% and β-turns ~9%) (Fig. 3A), ~22% corresponds to random structures and ~17% to α-helix.

At pH 5.0, there was a marked increment of 1654 cm^−1^ component (~12%) with a concomitant detriment of the 1630 cm^−1^ component compared to the components determined at pH 7.4 (Fig. 3B). These changes indicated that, at acidic pH, the β sheet content of apoB100 was diminished and the structural changes occurring in the protein result in a significant increase in the α-helix structure. Additionally, the component appearing at 1617 cm^−1^ was slightly augmented at pH 5.0, indicating higher β-strands content. On the other hand, the component assigned to random or unordered structures that appeared at ~1642 cm^−1^ decreased moderately at pH 5.0 compared to pH 7.4, indicating a more ordered structure (Fig. 3).

In order to corroborate whether these observed structural changes were stable or evolve over time, the acidified sample was incubated for 24 h at 4 °C before next spectrum recording. After that, neutral pH (7.4) was restituted by dialysis. The results of amide I band decomposition shown in Table 1 indicate that the secondary structure content of apoB100 after pH restitution is very similar to that observed at pH 7.4. These results reveal that changes induced by incubating LDL at pH 5.0 are reversible and characterized by an increment in the α-helix component and decreased β-sheet structure content even after 24 h incubation.

**Figure 4:**
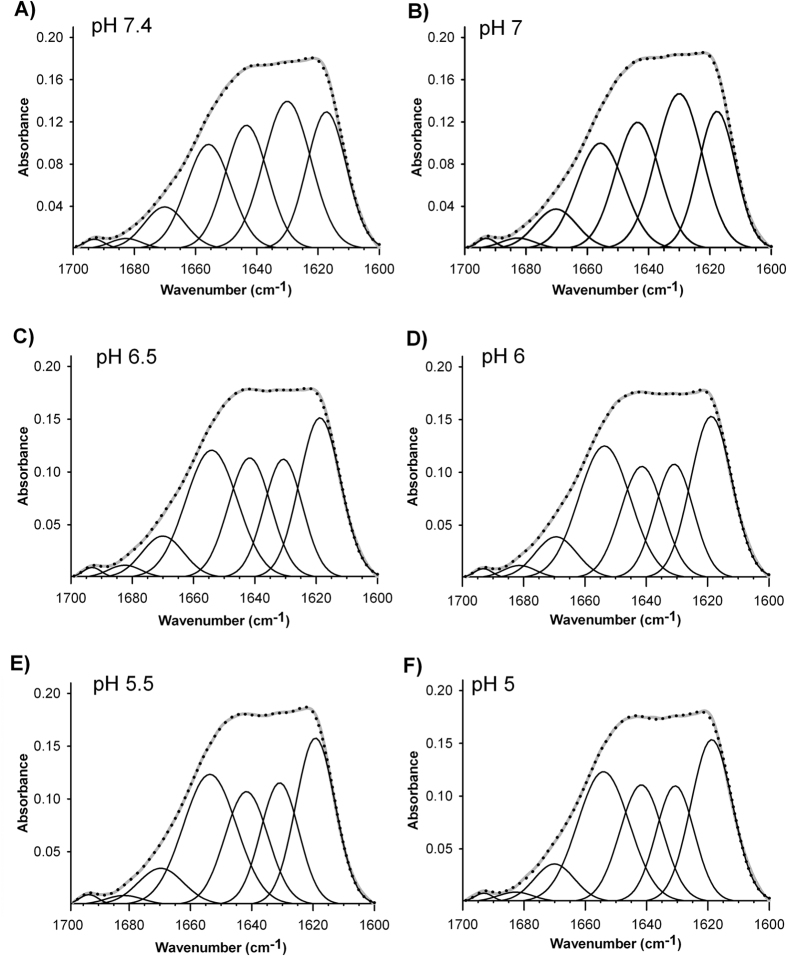
Transition from β-sheet to α-helix structure occurring as consequence of pH acidification. (**A**) pH 7.4, (**B**) pH 7.0, (**C**) pH 6,5, (**D**) pH 6.0, (**E**) pH 5,5, (**F**) pH 5.0. The spectra were obtained in D_2_O buffer at 37 °C and data processed as described in Materials and Methods.

**Figure 5:**
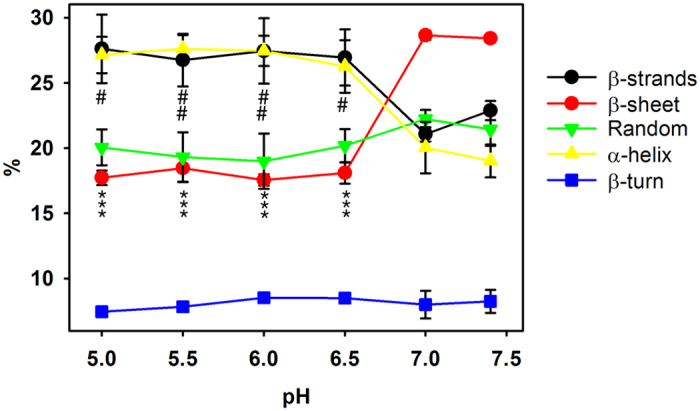
Percentage values of apoB100 secondary structure content at pH intervals (7.4 → 5.0). The spectra were obtained in D_2_O buffer at 37 °C and data processed as described in Materials and Methods. Data are mean ± S.D. (n = 3). All measurements were performed independently 3 times and levels of significance were determined by a two-tailed Student’s t-test. ***P < 0.001 when compared β -sheet percentage content at different pH with data obtained at pH 7.4. ^#^P < 0.05; ^##^P < 0.01 when compared α -helix percentage content at different pH with data obtained at pH 7.4.

We next studied the transition between β-sheet/α-helix structure occurring as consequence of pH acidification. Therefore, infrared spectra of apoB100 were recorded at pH increments of 0.5, ranging from pH 7.4 to pH 5.0. As shown in Fig. 4, the mayor transition from β-sheet to α-helix structure content occurs at pH 6.5 (Fig. 4A–C). Then, the α-helix content is slightly increased in detriment of β sheet content until pH 5.0 (Fig. 4D–F). The percentage values of beta/alpha structure content illustrating the transition at pH intervals (7.4 → 5.0) are shown in Fig. 5.

### Thermal stability of apoB100 LDL and lipid moiety at pH 7.4 and pH 5.0

**Figure 6:**
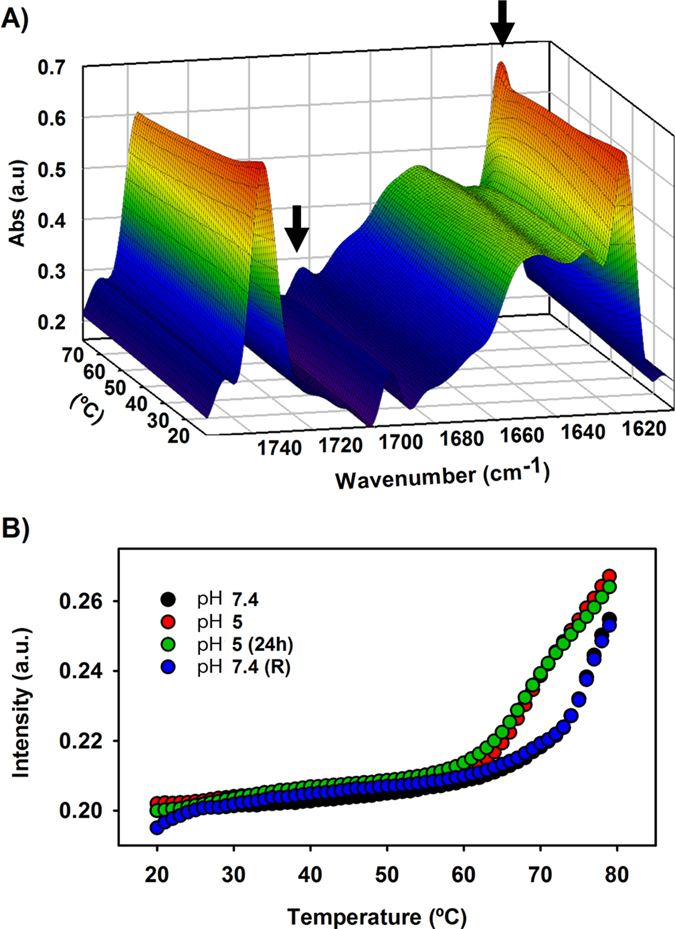
Thermal stability of apoB100 LDL and lipid moiety. (**A**) deconvoluted three-dimensional spectra of LDL at pH 7.4. LDL was heated from 20 to 80 °C at 1 °C increment per minute and the changes occurring in the protein structure were monitored by following the spectral band patterns at increasing temperatures as described in Materials and Methods. Aggregation occurring as a consequence of thermal denaturation, with bands appearing together at 1682 and 1617 cm^−1^ is indicated by black arrows. (**B**) Intensity of the 1682 cm^−1^ band as a function of temperature in LDL incubated at pH 7.4, pH 5.0, LDL incubated for 24 h at pH 5.0 and LDL after pH neutral restitution. The spectra were obtained in D_2_O buffer at 37 °C and data processed as described in Materials and Methods.

Despite changes occurring in the secondary structure of apoB100 at acidic pH are reversible, LDL stability may be altered due to tertiary structure modifications that are not totally restituted after pH neutralization. In order to gain knowledge on this possibility, we have determined the temperature at which aggregation bands appear at both pH 7.4 and 5.0 or after neutral pH restitution (5.0 → 7.4). The temperature shift can be taken as a change in apoB100 stability^26^. Figure 6A shows the deconvoluted three-dimensional spectra of LDL at pH 7.4, aggregation occurring as a consequence of thermal denaturation, with bands appearing together at 1682 and 1617 cm^−1^ is indicated by black arrows. Considering the 1682 cm^−1^ band as one of the representative indicators of aggregation and denaturalization, we next monitored the intensity of the 1682 cm^−1^ band as a function of temperature (Fig. 6B). The data show that at pH 7.4, band peaks showing protein aggregation are detected above 80 °C (Fig. 6B), similarly, at pH 5.0 the band intensity remains essentially identical up to 60 °C. However, from 60 °C the intensity begins to increase, indicating the first steps of aggregation that is an irreversible process. It is interesting to note that, after neutral pH restitution, the aggregation bands appear at similar temperatures than those of LDL at pH 7.4, indicating that tertiary structure alterations of apoB100 occurring at pH 5.0 are totally reversible (Fig. 6B). A decreased stability of apoB100 at pH 5.0 can be shown by the shift of the melting temperature (T_m_) caused by acidification which was found to be of ~10 °C (T_m_ at pH 5.0 = 70 °C and T_m_ at pH 7.4 = 80 °C, Fig. 6B). In addition, pH reversion allows recovery of protein stability (Fig. 6B).

In a similar way, intensity changes in absorption of cholesterol esters (1736 cm^−1^) and triglycerides (1750 cm^−1^) were also analyzed to study thermal stability of LDL lipid moiety. Below 30 °C, the thermal behavior of both lipids showed a slight different tendency suggesting a reduced lipid packing within LDL core, which has been previously described as a reversible phase transition^27^. The similar thermotropic behavior of these lipids at both pH indicates that pH changes did not affect to the lipid core of the LDL (data not shown).

## Discussion

It is well established that apoB100 binds with high affinity to the LDLR, *via* a ligand-binding domain consisting of seven cysteine-rich repeats (LR1–LR7)^28^. In the acidic environment of the endosome, the affinity of the LDLR β-propeller for LDLR cysteine-rich repeats is higher than for LDL and, the β -propeller functions as an alternative substrate for the LDLR ligand-binding domain^7^. Consequently, a conformational switch from an open (ligand-active) to a closed (ligand-inactive) LDLR conformation occurs, with a concomitant LDL release^4^,^29^. Molecular mechanisms occurring in LDLR at acidic pH are well known^4^,^6^,^7^, however, little attention has been paid to changes occurring in LDL, mainly because its structural complexity and the huge molecular size of apoB100. This limitation has led to the belief that LDL has a mere passive role in the dissociation process, mainly driven by conformational changes of LDLR at acidic pH^4^,^30^,^31^.

In the present work we have determined by DLS and NS-EM that size and morphology of LDL is not modified by pH acidification. In addition, the IR analysis performed in this work shows a conformational change of apoB100 that results in a higher α-helical and decreased β-sheet content at acidic pH compared to neutral pH. Furthermore, the ascertained reversibility of the apoB100 structure after neutral pH restitution suggests an active role of LDL in the dissociation process and evidences that apoB100 conformation is pH specific.

The structural modifications adopted by apoB100 at acidic pH may have a direct influence on the dissociation of the LDL/LDLR complex either by promoting or assisting a faster dissociation from the LDLR. Further studies should be performed to elucidate if LDL conformational changes at endosomal milieu are sufficient for the dissociation of LDL/LDLR complex or act coordinately with those occurring in LDLR. Accordingly, the appropriate LDLR structural modifications to adopt the closed conformation required for recycling may also be facilitated. It has been described that pH lowering favours dissociation of LDL/LDLR complex by reducing the affinity of LDLR- LR5 for apoB100^33^ and unfolding of the LDLR-LR5 repeat^33^. Binding analysis of LR5 repeat to mimicking peptides of LDL site B (exposed residues 3356–3368 located on the LDL surface)^34^ or LDL site A (an additional proposed binding site comprising residues 3143–3155)^34^,^35^,^36^ leads to consider a sequential binding model in which site B binds first to LDLR^32^. Then, re-organization in the LDL leads to a greater exposure of site A, allowing recognition of this site by another LR in the receptor^32^. In accordance with this model, the structural changes occurring upon acidification in apoB100 determined in this work may affect somehow or directly modify one of the recognition sites in apoB100 further displacing the equilibrium towards dissociation of LDL/LDLR complex. Alternatively, the whole apoB100 ligand binding domain may be affected by the structural rearrangement occurring at acidic pH, leading to the same effects described above.

In the present work we also have analyzed the effect of pH acidification on lipoprotein particle size and morphology by DLS and electron microscopy. LDL particles consist of a single copy of an apoB100 molecule wrapped around the surface of the particle composed of a monolayer of phospholipids, sphingomyelin and unesterified cholesterol molecules^11^,^37^. In the proposed belt-and-bow model, apoB100 makes a full turn around the particle, with a loose C-terminal part crossing over and forming a bow^38^ following a meandering path^10^. ApoB100 also penetrates partially the phospholipids monolayer reaching the outer core of the particle and interacts with the lipids of the deeper layer of LDL as well^10^,^39^. In addition, apoB100 is by necessity very flexible, as it must continually adapt its conformation and length to decreasing particle size during the lipolytic cascade from VLDL to LDL^40^. Here we show that neither particle size and morphology nor lipidic microenvironment of LDL is modified by pH acidification, indicating that structural modification occurring in apoB100 upon acidification does not alter physical properties of the particle with the protein matched to the morphology imposed by the lipid moiety of LDL. It has been previously shown that at different pH, the topology of loops and turns is altered leading to changes in the forces that stabilize the strands and the tertiary structure of the proteins^26^.

There is strong evidence that the acidic microenvironment in the arterial intima has a direct role in the development of atherosclerosis, characterized by the extra- and intra-cellular accumulation of lipoprotein-derived lipids. Acidity enhances processes such as proteolytic, lipolytic, and oxidative modifications of LDL and other apoB-containing lipoproteins^41^,^42^. Therefore, the conformational changes induced on apoB structure by acidic environment presented in this work could have special relevance in the pathophysiology of atherosclerosis as the association with LDLR would be reduced, what would increase LDL time residence in acidic compartment favouring modifications occurring within the atherosclerotic lesions^43^. It would be interesting to determine the changes occurring in other apoB100 containing lipoproteins such VLDL and IDL, however IR spectroscopy can not discriminate among the apoproteins contained in these lipoproteins and consequently structural information can not be specifically assigned to apoE, apoC2, apoC3 or apoB-100.

In conclusion, the data presented here show that pH acidification results in a different secondary structure content of apoB100 suggesting a more active role of apolipoprotein in the LDLR/LDL releasing process that occurs upon endosomal acidification. It is also remarkable the reversibility of the structural modifications determined after neutral pH restitution, which indicates, that apoB100 conformation is pH specific. The obtained results emphasize a more dynamic LDL/LDLR dissociation process than previous ascertained and provide new structural insights into the LDL/LDLR interactions than can occur at endosomal low-pH milieu.

## Additional Information

**How to cite this article**: Fernández-Higuero, J. A. *et al*. Structural changes induced by acidic pH in human Apolipoprotein B-100. *Sci. Rep.* **6**, 36324; doi: 10.1038/srep36324 (2016).

**Publisher’s note:** Springer Nature remains neutral with regard to jurisdictional claims in published maps and institutional affiliations.

## Figures and Tables

**Table 1:** Mean percent values of the secondary structure components of LDL at pH 7.4, 5.0 or after neutral pH restitution.

Structure	pH 7.4	pH 5.0	pH 5.0 (24 h)	pH 5.0 → 7.4
β -Turn	9.4 ± 0.4	8.2 ± 0.6	7.8 ± 0.4	9.6 ± 0.3
α – helix	17.0 ± 0.9	28.6 ± 0.9**	29.6 ± 0.7***	17.5 ± 0.8
Random	23.5 ± 0.8	21.8 ± 0.4	22.2 ± 0.8	22.9 ± 0.6
β – Sheet	28.2 ± 0.2	17.3 ± 0.7**	16.6 ± 0.7**	28.1 ± 0.1
β – Strand	21.8 ± 0.1	24.0 ± 0.2	23.8 ± 0.4	21.9 ± 0.2

Data are mean ± S.D. (n = 3). All measurements were performed independently 3 times and levels of significance were determined by a two-tailed Student’s t-test. **P < 0.01; ***P < 0.001 when compared data obtained at pH 5.0 with data obtained at pH 7.4.
